# Combination of culture-independent and culture-dependent molecular methods for the determination of bacterial community of *iru*, a fermented *Parkia biglobosa* seeds

**DOI:** 10.3389/fmicb.2012.00436

**Published:** 2013-01-07

**Authors:** Gbenga A. Adewumi, Folarin A. Oguntoyinbo, Santosh Keisam, Wahengbam Romi, Kumaraswamy Jeyaram

**Affiliations:** ^1^Department of Microbiology, Faculty of Science, University of LagosAkoka, Lagos, Nigeria; ^2^Microbial Resources Division, Institute of Bioresources and Sustainable Development, Takyelpat Institutional AreaImphal, Manipur, India; ^3^Department of Food Science and Technology, College of Food Sciences, Bells University of TechnologyOta, Nigeria

**Keywords:** *iru*, fermentation, safety, *Bacillus*, DGGE

## Abstract

In this study, bacterial composition of *iru* produced by natural, uncontrolled fermentation of *Parkia biglobosa* seeds was assessed using culture-independent method in combination with culture-based genotypic typing techniques. PCR-denaturing gradient gel electrophoresis (DGGE) revealed similarity in DNA fragments with the two DNA extraction methods used and confirmed bacterial diversity in the 16 *iru* samples from different production regions. DNA sequencing of the highly variable V3 region of the 16S rRNA genes obtained from PCR-DGGE identified species related to *Bacillus subtilis* as consistent bacterial species in the fermented samples, while other major bands were identified as close relatives of *Staphylococcus vitulinus*, *Morganella morganii*, *B. thuringiensis*, *S. saprophyticus*, *Tetragenococcus halophilus*, *Ureibacillus thermosphaericus*, *Brevibacillus parabrevis*, *Salinicoccus jeotgali*, *Brevibacterium* sp. and uncultured bacteria clones. *Bacillus* species were cultured as potential starter cultures and clonal relationship of different isolates determined using amplified ribosomal DNA restriction analysis (ARDRA) combined with 16S–23S rRNA gene internal transcribed spacer (ITS) PCR amplification, restriction analysis (ITS-PCR-RFLP), and randomly amplified polymorphic DNA (RAPD-PCR). This further discriminated *B. subtilis* and its variants from food-borne pathogens such as *B. cereus* and suggested the need for development of controlled fermentation processes and good manufacturing practices (GMP) for *iru* production to achieve product consistency, safety quality, and improved shelf life.

## Introduction

Fermented vegetable protein seeds used for condiments production in West Africa include African locust bean [*Parkia biglobosa* (Jacq. Benth)], melon seeds [*Citrullus vulgaris* (Schrad)], castor oil seeds (*Ricinus communis*), fluted pumpkin seeds (*Telfaria occidentale*), African yam bean (*Stenophylis stenocarpa*), cotton seeds [*Gossypium hirsitium* (L.)], roselle seeds (*Hibiscus sabdariffa*), and baobab seeds [*Adansonia digitata* (L.)] (Odunfa and Oyewole, 1998; Ouoba et al., 2008; Parkouda et al., 2010). Condiments constitute a significant proportion of African diets where they serve as flavorsome and culinary components in various dishes (Achi, 2005). They include *iru* or *dawadawa* in Nigeria and Ghana; *soumbala*, *bikalga*, and *maari* in Burkina Faso; *afitin* and *sonru* in Benin Republic; *nététou* in Senegal; *kinda* in Sierra Leone; *dawadawa botso* in Niger Republic; *datou* in Mali; *mbuja* in Cameroon and *furundu* in Sudan (N'Dir et al., 1994; Ouoba et al., 2008, 2010; Parkouda et al., 2010).

Though, the vegetable seeds used for the production of these condiments differ considerably from one region to another (Achi, 2005). Nonetheless, the production process is generally characterized by spontaneous solid substrate alkaline fermentation with increase in pH as a result of extensive hydrolysis of the proteins into peptides, amino acids and ammonia, which favors the dominance of *Bacillus* spp. as the fermenting organisms (Kiers et al., 2000; Sarkar et al., 2002; Ouoba et al., 2003; Achi, 2005). The art is also carried out in exclusive uncontrollable environmental conditions thereby yielding products with variation in quality and organoleptic properties (Sanni, 1993; Steinkraus, 1997; Ouoba et al., 2007). This necessitates the need for the development of appropriate starter cultures to initiate fermentation for the production of consistent products with acceptable qualities.

The processing operations of African locust bean to produce *iru* involve cleaning and sorting, boiling, dehulling, and fermentation at ambient temperature (Oyeyiola, 1988). Investigations on the microbiology of *iru* using selective isolation and cultivation based on traditional culture-dependent methods including the biochemical changes that occur during fermentation have been documented (Odunfa, 1981, 1985; Omafuvbe et al., 2004). These techniques often fail to account for minor microbial populations; stressed and injured cells that are present in low number (Fleet, 1999), and have been observed not to give a complete representation of food microbial communities (Kesmen et al., 2012). Also, they are bias, unreliable and lack accurate detection of population dynamics and microbial diversity (Amann et al., 1995; Hugenholtz et al., 1998; Giraffa and Neviani, 2001; Jany and Barbier, 2008).

Culture-independent microbial techniques, such as PCR-denaturing gradient gel electrophoresis (DGGE) has proven much more reliable, fast, and economical in profiling complex bacterial community structure, dynamics and evolution of microbial populations of fermented foods (Giraffa and Neviani, 2001; Randazzo et al., 2002; Cocolin et al., 2007; Jany and Barbier, 2008). PCR-DGGE has been successfully employed to studying the microbial community structures and dynamics of fermented vegetal protein seeds such as *doenjang* and *meju* (Cho and Seo, 2007; Kim et al., 2009; Lee et al., 2010) as found in Asia. More recently, barcoded pyrosequencing of V1/V2 region of 16S rRNA gene was used in assessing the microbial community of *doenjang* (Nam et al., 2012). Meanwhile, there is dearth of information on the application of these techniques to profile microbial community of fermented vegetable foods in Africa.

Studies involving the use of both culture-dependent and culture-independent molecular methods for the identification of dominant bacterial species; accurate determination and detailed investigation of microbial composition of fermented food products have been reported (Pulido et al., 2005; Vilela et al., 2010; Alegría et al., 2012; Kesmen et al., 2012). In this study, we combined both culture-dependent and -independent molecular techniques as a polyphasic strategy to first understand and characterize the microbial community profile of *iru* produced by natural, uncontrolled fermentation process; secondly, to have a deeper insight into the bacterial diversity, which can be used to locate and identify the dominant bacteria with technological and functional potentials that can be further developed as autochthonous starter cultures to improve industrial production of condiments in W. Africa and guarantee product safety.

## Materials and methods

### Sample collection

Fermenting *P. biglobosa* during *iru* production were obtained from local producers as well as retail markets samples in southwest (Lagos, Ibadan, Abeokuta, Oyo, and Ado-Ekiti) and north central Nigeria (Kaduna and Ilorin). They were transported immediately into the laboratory with the aid of ice pack, stored in the refrigerator at 4°C and analyzed within few days of collection.

### Isolation of microorganisms

Samples of fermented *iru* were homogenized in 90 ml Ringer's solution. Serial dilutions were made and 0.1 ml of appropriate dilutions was spread plated on nutrient agar (Oxoid, Hampshire, UK). Plates were subsequently incubated at 37°C for 18–24 h. Bacterial colonies obtained were purified by repeated streaking to obtain pure cultures.

### Culture-dependent analysis

A total of 280 bacterial isolates were isolated from 20 *iru* samples and Reference bacterial strains obtained from Microbial Type Culture Collection (MTCC), India were subjected to genotypic characterization. Amplified ribosomal DNA restriction analysis (ARDRA) combined with PCR amplification of the 16S–23S rRNA gene internal transcribed spacer (ITS), ITS-PCR-restriction fragment length polymorphism (ITS-PCR-RFLP), and randomly amplified polymorphic DNA (RAPD-PCR) as previously described (Oguntoyinbo et al., 2010) were used for genotypic grouping, clonal relationship, and isolates diversity. The almost complete 16S rRNA genes of representative isolates from the clusters generated from dendrogram of gel fingerprints were amplified and sequenced as described previously (Jeyaram et al., 2010). Closest known identities of the 16S rRNA gene sequences were obtained from NCBI GenBank database.

### Culture-independent analysis

#### DNA extraction and PCR amplification

Two methods of bacterial genomic DNA described previously by Ercolini et al. (2003) and Rantsiou et al. (2005) were used to extract genomic DNA from 16 *iru* samples (no appreciable DNA was obtained from remaining four samples). The V3 region of the 16S rRNA gene was amplified using the set of primers 338f (5′-ACTCCTACGGGAGGCAGCAG-3′) and 518r (5′-ATTACCGCGGCTGCTGG-3′) (Sigma-Aldrich). A GC-rich clamp was attached to the forward primer as described by Muyzer et al. (1993). Five-microliters PCR amplified product was analysed on 2% agarose (Promega, USA) containing ethidium bromide (0.5 μg/ml) (E1510, Sigma-Aldrich), using 0.5× TBE buffer (45 mM Tris-borate, 1 mM EDTA, pH 8.0) with running conditions of 80 V for about 1 h. DNA fragments of about 240 bp obtained by comparing with PCR 100 bp low ladder (P1473, Sigma-Aldrich) was used for DGGE analysis.

#### DGGE analysis of fermented iru samples

DGGE was performed using a DCODE™ Universal Mutation Detection System (BIO-RAD, USA) following manufacturer's instructions. PCR amplicons including a DGGE marker designed and used as a reference DNA ladder, were separated in 8% polyacrylamide gels containing 1× TAE with denaturing gradients of 25–55% [100% denaturant corresponds to 7 M urea and 40% (v/v) formamide]. Electrophoresis was run initially at 20 V for 10 min and at a constant temperature of 60°C, and finally at 140 V for 5 h at 60°C. The gels were stained with ethidium bromide, visualized under UV light and documented using Gene Snap (PerkinElmer GELIANCE 200, USA).

#### PCR-DGGE gel images analysis

The DGGE gel images obtained were converted to densitometric profiles using the software TL120 v2006 (Phoretix 1D Advanced Software, NonLinear Dynamics, Newcastle, UK). The similarity between the DGGE lanes was investigated by constructing non-metric multidimensional scaling (non-metric MDS) scatter plots as described elsewhere (Boon et al., 2002; McOrist et al., 2008) using the software package P A S T (PAleontological STatistics, http://folk.uio.no/ohammer/past/). The Shannon–Weaver index of *H* diversity and *R* richness index were determined based on the number presence/absence in the fragments. The importance probability *Pi* and *H* were calculated using PAST (Shannon and Weaver, 1963).

#### Excision of DGGE bands and sequencing of amplicons

DGGE major bands of interest were excised from the polyacrylamide gels, eluted in 50 μl sterile deionized water (Milli Q, Millipore, Bangalore) and incubated overnight at 4°C as described by Cocolin et al. (2001). One microliter of each eluate was used as DNA template for PCR reamplification. PCR products originating from the excised bands were profiled and checked for quality in agarose; presence and position of the bands of interest in DGGE and comparison with parent DGGE profiles. Elution and reamplification were carried out at least twice or until a single band that co-migrate at the same position with the parent DGGE band is obtained. Sequences of major bands obtained from the DGGE gel fragments were compared with the NCBI GenBank database to determine closest relatives.

## Results

### Culture-based molecular approach

In the cultured analysis, similar colonial morphology and population profile ranging from 10^6^–10^7^ CFU/ml were obtained from fermented samples as well as final product. A total of 280 isolates selected for analysis by ARDRA restriction endonucleases especially *Cfo*I showed differentiation of the isolates into varying species, in particular, *B. subtilis* and *B. cereus* phylotypes. Further characterization with ITS-PCR-RFLP revealed intraspecies divergence among the *Bacillus* isolates. The gel data of ARDRA, ITS-PCR, ITS-PCR-RFLP, and RAPD-PCR were combined to generate polyphasic gel fingerprint. Representative of the major clusters obtained were identified by sequence data of 16S rRNA gene that identified two major bacilli groups; *B. subtilis* and *B. amyloliquefaciens* as the dominant, as well as *B. cereus* group as member of the cultivatable bacteria associated with fermented *P. biglobosa* cotyledon (Table 1).

**Table 1:** **Genomic characterization of *Bacillus* species isolated from *iru*, traditional fermented *Parkia biglobosa***.

**ARDRA groups**	**16S rRNA-RFLP profile (sizes in bp)**						**ITS-PCR sub- groups (sizes in bp)**	**ITS-PCR-RFLP sub-groups (sizes in bp)**	**No. of isolates**	**Representative strains**	**16S rRNA gene sequencing**		
	***Hae*III**	***Cfo*I**	***Dde*I**	***Hinf*I**	***Taq*I**	***Rsa*I**					**Closest known relative**	**Similarity**	**NCBI acc. no.**
**I**	^a^627, 473	908	508	581	573	525	**IA.**	**IA1.**	93	U4E BA	*B. subtilis*	99%	JN255703
	318	451	465	355	458	477	1065, 945,	502, 417, 339, 278, 260, 214,					
	156	248	237	317	349	429	869,	143, 136					
			150	177	214	149		**IA2.**	26	U104	*B. subtilis*	98%	JN165753.1
			122					506, 339, 280, 141					
**II**	622, 467	904	504	592	590	515	**IIA.**	**IIA1.**	1	U154B	*B. subtilis*	100%	JN255715
	314, 151	445,	381	362	412	470	1036, 845	485, 401, 322, 263, 247, 218,					
		243	280	324	361	417		138, 129					
			147	183	221	146		**IIA2.**	3	U191A	*B. subtilis*	97%	JN255725
			117					489, 329, 271, 231, 137					
								**IIA3.**	1	U233	*B. subtilis*	100%	JN255727
								494, 406, 330, 269, 255,					
								225, 127, 120					
								**IIA4.**	1	U122A	*B. subtilis*	99%	JN255710
								494, 329, 268, 223, 134					
								**IIA5.**	2	U213A	*B. pumilus*	99%	JN255726
								490, 339 259, 149		U158	*B. pumilus*	100%	JN255717
**III**	627, 472,	912	510	609	722	515	**IIIA.**	**IIIAl.**	62	U184B	*B.amylolique-faciens*	99%	JN255722
	318, 156	451	392	374	375	424	1057, 939, 863	504, 420, 343, 274,					
		247	286	335	232	147		259, 145, 137					
			236	190				**IIIA2.**	37	U79BA	*B.amyloliquefaciens*	98%	JN255707
			122					506, 424, 384, 369, 343,					
								274, 260, 147, 138					
**IV**	622, 475	414	518	581	570	498	**IVA.**	**IVAl.**	3	U186	*B. licheniformis*	98%	JN255724
	324, 156	326	288	344	395	459	1024, 845	496, 336, 272, 229, 142					
		228	277	307	345	409	**IVB.**	**IVB1.**	1	U126	*B. licheniformis*	100%	JN255711
		172	237	166	211	142	1044, 926,	489, 435, 414, 335, 271, 129					
			132				842, 339						
			115										
**V**	595, 477	567	516	943	600	494	**VA.**	**VA1.**	49	U175	*B. cereus*	96%	JN255721
	322, 161	417	287	312	481	411	1078, 827	506, 311, 168, 136		U243	*B. cereus*	92%	JN255728
		332	275	170	134	355							
		175	172			142							
			150										
			115										
**VI**	627, 477,	412	546	973	575	452	**VIA.**	**VIA1.**	1	U185B	*Brevibacillus formosus*	99%	JN255723
	228, 157	324	441	304	389	411	1359, 1161,	461, 355, 294, 262, 225, 205,					
		218	238	234	365	353	1103, 1020,	198, 193, 167, 157, 142, 133					
		172	167	167	196	131	910						
			120			107							

a *DNA fragment sizes*.

### Analysis of bacterial communities and diversity of iru using PCR-DGGE

**Figure 1:**
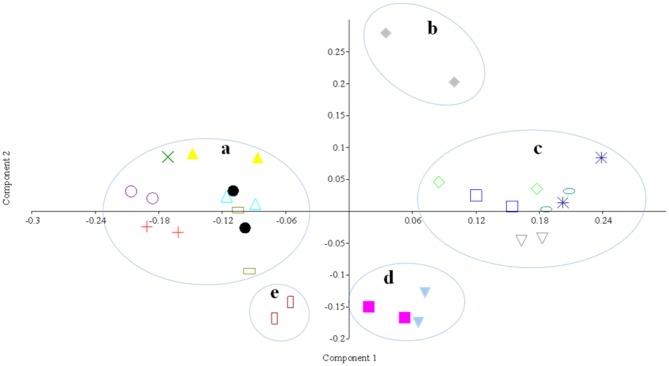
**Non-metric Multidimensional Scaling analysis of DGGE data Group (a): *iru* Abeokuta 2 southwest Nigeria (**
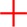
**); *iru* Abeokuta 3 southwest Nigeria (**
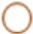
**); *iru* Lagos 1 southwest Nigeria (**
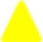
**); *iru* Lagos 2 southwest Nigeria (•); *iru* Ilorin 1 northcentral Nigeria (**
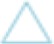
**); *iru* Ilori n 2 northcentral Nigeria (**
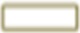
**); *iru* Ibadan 1 southwest Nigeria (**
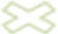
**)**. Group (b): *iru* Abeokuta 4 southwest Nigeria (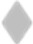). Group (c): *iru* Abeokuta 1 southwest Nigeria (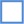); *iru* Abeokuta 5 southwest Nigeria (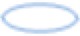); *iru* Abeokuta 8 southwest Nigeria (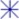); *iru* Oyo southwest Nigeria (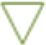); *iru* Ado-Ekiti southwest Nigeria (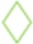). Group (d): *iru* Abeokuta 6 southwest Nigeria (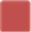); *iru* lbadan 2 southwest Nigeria (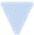). Group (e): *iru* Abeokuta 7 southwest Nigeria (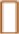).

The two genomic DNA extraction protocols- enzymatic and chemical proved to be efficient in terms of quantity, purity, and amplifiabiltiy, and thus produced DNA suitable for PCR amplification. The results of the PCR-DGGE gel fingerprints of the V3 region of amplified 16S rRNA gene showed identical DGGE patterns and high degree of similarity in DNA fragments with the two DNA extraction methods. The PCR-DGGE gel profiles were also used to assess the bacterial community profile; richness, biodiversity, and dominance indexes of naturally fermented *iru* samples obtained from different geographical locations in Nigeria. The species richness index (*R*) determined was highest for DGGE profiles of *iru* samples from Ilorin (*R* 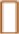 19), thus exhibiting the highest number of bands/bacterial species than those of Lagos (16 < *R* < 18); Ado-Ekiti (*R* 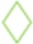 16); Ibadan (11 < *R* < 14); Oyo (*R* 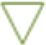 10) and Abeokuta (9 < *R* < 17) (Table 2). Bacterial diversity index (*H*) calculated on the basis of number of bands on a gel track was also highest for *iru* samples from Ilorin (*H* 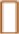 2.92) with the lowest being Abeokuta (2.19 < H < 2.80) (Table 2). Similarity and variation among DGGE gel patterns of the various fermented condiments were established based on combined analysis of Dice similarity coefficient and nMDS. The results obtained showed that the 16 *iru* samples analysed clustered into five groups—a, b, c, d, and e (Figure 1). Noticeable similarity can be said to exist in the bacterial community structure of the different samples of *iru* obtained in the two geographical zones under study (southwest and north central Nigeria). Apart from two *iru* samples from Lagos south western Nigeria, that clustered into the same group “a” variation was observed among samples within the same geographical location especially southwest Nigerian samples.

**Table 2:** **Species richness estimates (*R*) and Shannon's index of diversity (*H*) of DGGE profiles of fermented *iru* samples obtained from various geographical locations in Nigeria**.

**Samples**	***R***	***H***
*iru* Abeokuta 1	12	2.44
*iru* Abeokuta 2	13	2.57
*iru* Abeokuta 3	17	2.80
*iru* Abeokuta 4	12	2.49
*iru* Abeokuta 5	11	2.34
*iru* Abeokuta 6	11	2.35
*iru* Abeokuta 7	15	2.70
*iru* Abeokuta 8	9	2.19
*iru* Lagos 1	18	2.88
*iru* Lagos 2	16	2.74
*iru* Ilorin 1	19	2.92
*iru* Ilorin 2	19	2.92
*iru* Ibadan 1	11	2.40
*iru* Ibadan 2	14	2.60
*iru* Oyo	10	2.25
*iru* Ado-Ekiti	16	2.77
Mean *iru* Abeokuta (±STDEV)	12.50 ± 2.35	2.49 ± 0.19
Mean *iru* Lagos (±STDEV)	17.00 ± 1.00	2.81 ± 0.05
Mean *iru* Ilorin (±STDEV)	19.00 ± 0.00	2.92 ± 0.00
Mean *iru* Ibadan (±STDEV)	12.50 ± 1.50	2.50 ± 0.10

### Identification of major bacterial PCR-DGGE bands

**Figure 2:**
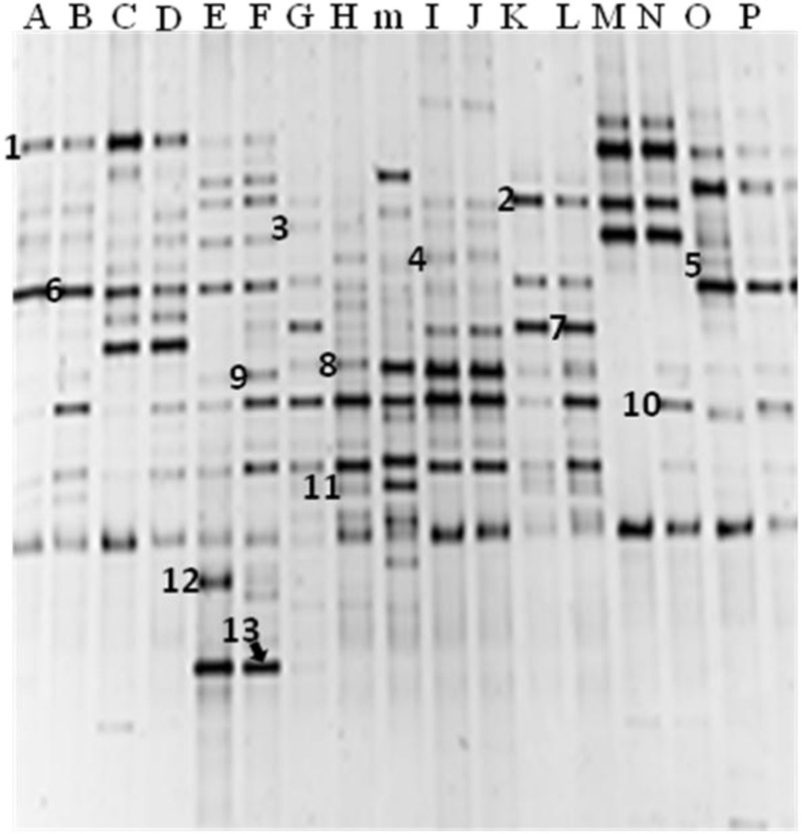
**DGGE profiles of PCR-amplified 16S rRNA gene fragments of sixteen (16) fermented *iru* samples showing major bacterial amplicons.** Samples A: *iru* Oyo; B: *iru* Lagos 1; C: *iru* Ilorin 2; D: *iru* Ilorin 1; E: *iru* Ibadan 1; F: *iru* Ado-Ekiti; G: *iru* Abeokuta 2; H: *iru* Abeokuta 1; I: *iru* Ibadan 2; J: *iru* Abeokuta 3; K: *iru* Abeokuta 4; L: *iru* Abeokuta 5; M: *iru* Abeokuta 6; N: *iru* Lagos 2; Î: *iru* Abeokuta 7; P: *iru* Abeokuta 8; “m” is DGGE reference DNA ladder. Bands 1: *Staphylococcus vitulinus;* 2: *Morganella morganii;* 3: *Bacillus thuringiensis;* 4: *B. licheniformis;* 5: *S. saprophyticus;* 6: Uncultured bacterium clone; 7: *Tetragenococcus halophilus;* 8: *Ureibacillus thermosphaericus;* 9: *Bacillus* sp.; 10: *B. subtilis; 11: Brevibacillus parabrevis;* 12: *Salinicoccus jeotgali;* 13: *Brevibacterium* sp.

DNA sequencing was carried out on the major bacterial bands. The closest known identities, percentage similarity and frequency of occurrence of each band in the fermented condiments are as shown in Table 3. Among them all, *B*. *subtilis* occurred most frequently (band 10), Figure 2). It was found to be present in 94% of the *iru* samples, which confirms its consistency, dominance, and viability in the fermented condiments. *B. licheniformis* and *Brevibacillus* species previously isolated in the culture-dependent study were also found present in at least a sample of the fermented condiment (Table 3). Other bands identified include close relatives of *S. vitulinus*, *Morganella morganii*, *B. thuringiensis*, *S. saprophyticus*, *T. halophilus*, *Ureibacillus thermosphaericus*, *Salinicoccus jeotgali*, *Brevibacterium* sp., and uncultured bacteria clones.

**Table 3:** **Identities of major bacterial bands excised from PCR-DGGE gels of fermented *Parkia biglobosa* seeds**.

**Bands**	**Source/locations^a^**	**16S rRNA gene closest known relative**	**% Similarity**
1	Oyo (1), Abeokuta (4), Ado-Ekiti (1), Lagos (1), Ibadan (1), Ilorin (2)	*Staphylococcus vitulinus*	98
2	Ado-Ekiti (1), Ibadan (2), Abeokuta (4), Lagos (2), Ilorin (2)	*Morganella morganii*	98
3	Abeokuta (2), Ibadan (1), Lagos (1)	*Bacillus thuringiensis*	99
4	Abeokuta (2), Ibadan (1), Ilorin (1)	*B*.* licheniformis*	98
5	Oyo (1), Abeokuta (1), Lagos (1), Ilorin (1)	*S*.* saprophyticus*	99
6	Oyo (1), Abeokuta (7), Ado-Ekiti (1), Lagos (2), Ilorin (2)	Uncultured bacterium clone	97
7	Abeokuta (3), Ibadan (1), Lagos (1)	*Tetragenococcus halophilus*	95
8	Oyo (2), Ibadan (2), Abeokuta (1), Lagos (1)	*Ureibacillus thermosphaericus*	100
9	Ado-Ekiti (1), Ibadan (1), Abeokuta (1), Lagos (1)	*Bacillus* sp.	98
10	Oyo (1), Abeokuta (7), Ado-Ekiti (1), Ibadan (2), Lagos (2), Ilorin (2)	*B. subtilis*	99
11	Abeokuta (1)	*Brevibacillus parabrevis*	97
12	Ibadan (1), Abeokuta (1)	*Salinicoccus jeotgali*	99
13	Ado-Ekiti (1), Lagos (1)	*Brevibacterium* sp.	97

*Bands are as shown in Figure 2*.

a *Values in parenthesis represent number of fermented iru samples, with total number of iru sampled being 16*.

## Discussion

In W. Africa traditional fermented condiments form an important component of the dietary protein requirement of the people, where they are added generously to various dishes to supplement protein intake, improve nutritive value, and impart sensory attributes. This study combined molecular techniques to reveal microbiota of traditional fermented condiments sample tested at different fermentation time regimes with consideration for geographical spread in Nigeria. This is aimed at future objectives of developing predictable process through the use of identified bacteria that can be applied as starter cultures to dominate the fermentation, impact desirable biochemical changes and improve safety quality.

The results of culture-dependent analysis and 16S rRNA gene sequencing showed that *iru* is composed of clonally related *Bacillus* species identified as *B*. *subtilis*, *B*. *amyloliquefaciens*, *B*. *cereus*, *B*. *licheniformis*, and *Brevibacillus formosus* in order of frequency of incidence. Also, the combination of ARDRA, ITS-PCR, ITS-PCR-RFLP, and RAPD-PCR revealed high degree of diversity among the *Bacillus* isolates and identified the dominant species to be *B*. *subtilis* and *B*. *amyloliquefaciens*. The results clearly showed sub-typing of autochthonous clonally related species of bacilli and genetically identified dominant groups, this is in agreement with previous reports in W. Africa and India during fermentation of similar vegetable proteins studies (Sarkar et al., 2002; Ouoba et al., 2004; Jeyaram et al., 2008; Kim et al., 2010; Oguntoyinbo et al., 2010; Parkouda et al., 2010).

Culture-independent techniques have been previously used to describe microbiota of fermented foods in Africa (Humblot and Guyot, 2009; Oguntoyinbo et al., 2011). Although none of this studies reported microbiota of fermented vegetable protein used as condiments. In this study, during profiling of bacterial community of fermented *iru*, two methods of genomic DNA extraction were employed. No significant differences in the DNA yields, fragments and DGGE band patterns were observed. Mafra et al. (2008) reported similar findings in a study involving comparison of four DNA extraction methods for downstream processing, comparison such as this guarantees better microbiome profiling. Results of PCR-DGGE gel analysis of *iru* sampled from Ilorin, north central Nigeria had the highest number of bacterial species than others, including the highest bacterial diversity index; the variable production process could possibly be responsible for this observation. The intraspecific variation observed in the fermented *iru* samples as predicated by Dice similarity coefficient and nMDS is an indication that the bacterial community profile of this condiment is diverse; having grouped into distinct clusters with varying diversity indexes. *Iru* from different geographical zones were produced from the same substrate *P. biglobosa* seeds but variable fermentation periods. Therefore, diversity observed in the microbial communities could be due to difference in fermentation conditions, which is also spontaneous and uncontrolled, without the use of any known specific isolate as starter culture. Other workers have also reported similar variation in the microbial composition of naturally fermented food materials produced using same substrate (Ampe and Miambi, 2000; Oguntoyinbo et al., 2011).

PCR-DGGE bands sequencing identified *B*. *subtilis* as the major bacterial species associated with *iru*, which is in agreement with the result of culture-based PCR analysis. Earlier studies using traditional culture-dependent methods and biochemical characterization have also reported the dominance of *B*. *subtilis* during *iru* fermentation (Antai and Ibrahim, 1986; Odunfa and Oyewole, 1986). Other DGGE bands corresponded to potential food-borne pathogens and contaminants such as *S. vitulinus*, *S*. *saprophyticus*, *B*. *thuringiensis*, *Tetragenococcus halophilus*, etc. Of interest is *T*. *halophilus*, a halophilic lactic acid bacterium, predominantly found in fermented foods with high salt concentration such as soysauce, *miso* and *doenjang* (Tanasupawat et al., 2002; Onda et al., 2003; Kim et al., 2009). The source of this bacterium in *iru* can somewhat be attributed to the salt added as a preservative at the end of the fermentation process. The detection of *B.cereus*, *B*. *thuringiensis*, and *Staphylococcus* species clearly raises doubt regarding the microbiological safety of this fermented condiment. Information on the toxigenic potential of food-borne pathogen in traditional African fermented foods exist (Oguntoyinbo and Oni, 2004; Abriouel et al., 2007; Oguntoyinbo et al., 2010).

In conclusion, the results indicated that combination of culture-dependent and culture-independent techniques can be used to profile bacterial microbiota, examine dynamics and diversity of naturally fermented food. On the overall, this study showed that bacterial composition of *iru* is significantly more diverse than earlier reported and justified the need to develop process across production sites to facilitate safety quality and consistency. This study confirmed dominance of *Bacillus* species and occurrence of other bacteria with pathogenic trait and unknown functions. Condiments can support dietary need of the people if better understanding of the functional roles of all members of the community during fermentation can be determined, this will support development of predictable process, eliminate variation and inconsistency.

### Conflict of interest statement

The authors declare that the research was conducted in the absence of any commercial or financial relationships that could be construed as a potential conflict of interest.
